# Supplementary material for: Combining hypoxia-activated prodrugs and radiotherapy in silico: Impact of treatment scheduling and the intra-tumoural oxygen landscape
Source: PLoS Comput Biol. 2020 Aug 3;16(8):e1008041. doi: 10.1371/journal.pcbi.1008041 (PMC7425994; doi:10.1371/journal.pcbi.1008041)
Supplement: S1 Text — (PDF) [file pcbi.1008041.s001.pdf]

Combining hypoxia-activated prodrugs and radiotherapy *in silico*: Impact of treatment scheduling and the intra-tumoural oxygen landscape

Sara Hamis[1,2], Mohammad Kohandel[3], Ludwig J Dubois[4], Ala Yaromina[4], Philippe Lambin[4], *Gibin G Powathil[2].

1 School of Mathematics and Statistics, University of St Andrews, St Andrews, Scotland.

2 Department of Mathematics, College of Science, Swansea University, Swansea, Wales, United Kingdom.

3 Department of Applied Mathematics, University of Waterloo, Waterloo, Canada.

4 The M-Lab, Department of Precision Medicine, GROW – School for Oncology and Developmental Biology, Maastricht University, Maastricht, The Netherlands.

S1 Text: Supporting Information

S1 Text-1a: Complement to Fig 12 – *HAP-IR treatment scheduling impacts HAP efficacy in sufficiently hypoxic tumours.*

S1 Text-1b: Complement to Fig 12 – *The* $[pO2]_{50}$ *value influences scheduling outcomes.*

S1 Text-2: Complement to Fig 13 – *HAPs enhance radiotherapy effects in sufficiently hypoxic tumours.*

S1 Text-3: Complement to Fig 15 – *The intra-tumoural oxygen landscape impacts HAP efficacy.*

S1 Text-4: *Pseudo-code flowchart.*

S1 Text-1a: Complement to Fig 12

Figs A and B show that the scheduling-experiment, with results provided in Fig 12 in the main manuscript, are qualitatively the same if a damaged cell is instantly removed from the lattice (Fig A) or if a damaged cell is moved from the lattice after a time period corresponding to its doubling time (Fig B).

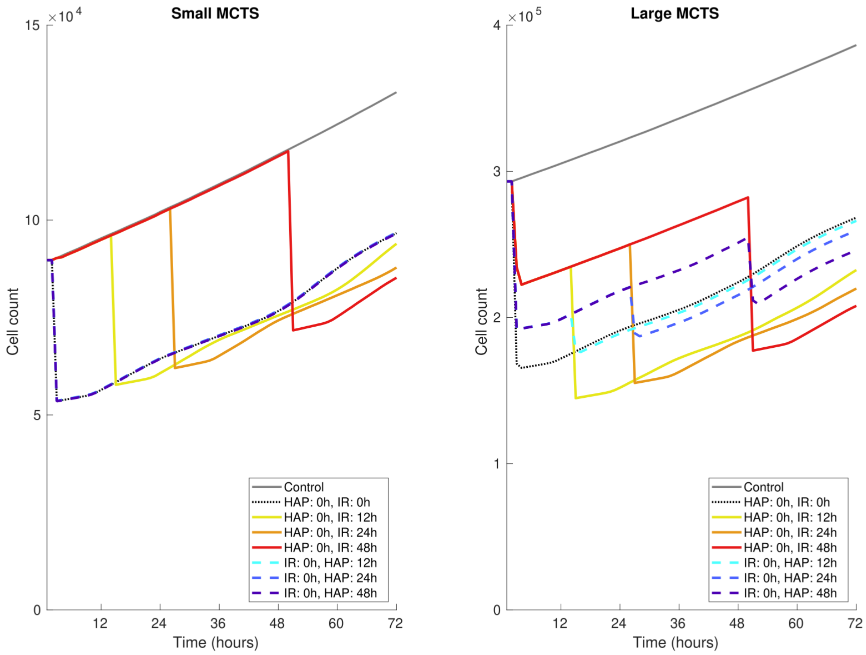

Fig A. Scheduling of HAP-IR combination treatments, complement to Fig 12 in the main manuscript. Cells are removed from the lattice instantaneously after the lethal event occurred.

S1 Text-1b: The $[pO_2]_{50}$ value influences scheduling outcomes

The $[pO_2]_{50}$ parameter value, denoting the oxygen value yielding 50% HAP-to-AHAP hourly bioreduction (see Fig 5 in the main manuscript), impacts the efficacy of various HAP-IR combination therapy schedules. To demonstrate this, Figs C (left) and C (right) respectively show the cell count over time when the 'Small' and 'Large' tumour (illustrated in Fig 8 in the main manuscript) are subjected to various HAP-IR schedules.

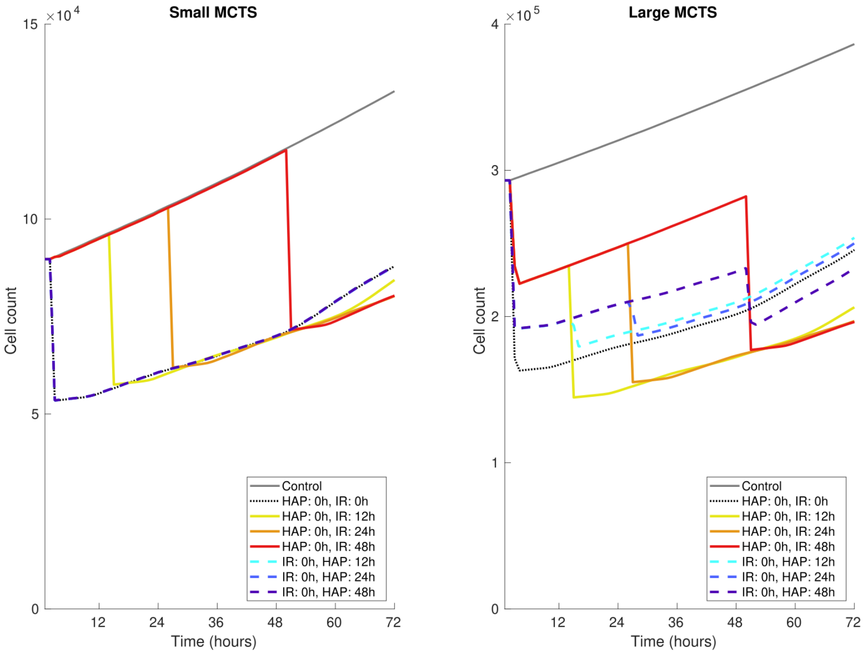

Fig B. Scheduling of HAP-IR combination treatments, complement to Fig 12 in the main manuscript. Cells are removed from the lattice after a time corresponding to their doubling time (τ_i) post the lethal event.

In Fig C, the $[pO_2]_{50}$ value has been increased with a factor of 5 from its original value used in the *in silico* experiments described in the manuscript (see results in Fig 12).

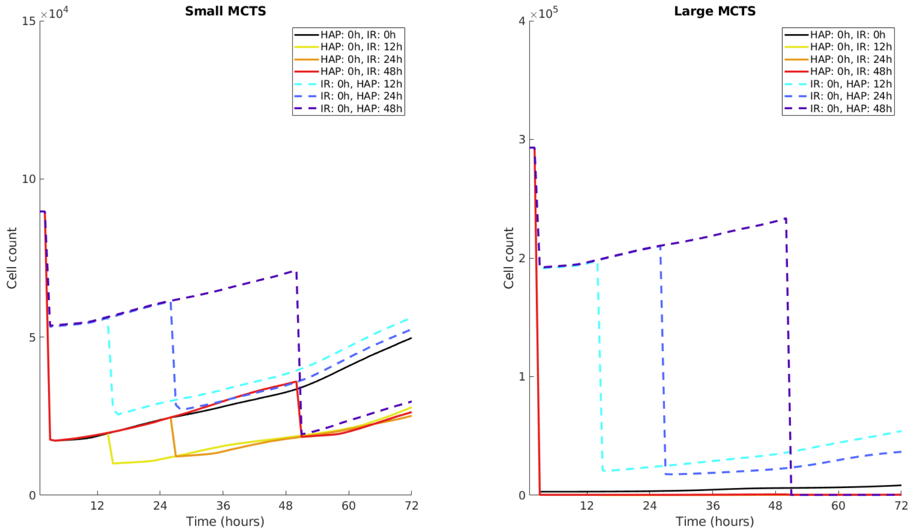

Fig C. Scheduling of HAP-IR combination treatments, complement to Fig 12 in the main manuscript. The $[pO_2]_{50}$ value is 5 times larger than in the original *in silico* experiments described in the manuscript (see Fig 12).

S1 Text-2: Complement to Fig 13

Figs D and E show that the experiment that investigates if HAPs act as radiotherapy enhancers, with results provided in Fig 13 in the main manuscript, are qualitatively the same if a damaged cell is instantly removed from the lattice (Fig D) or if a damaged cell is moved from the lattice after a time period corresponding to its doubling time (Fig E).

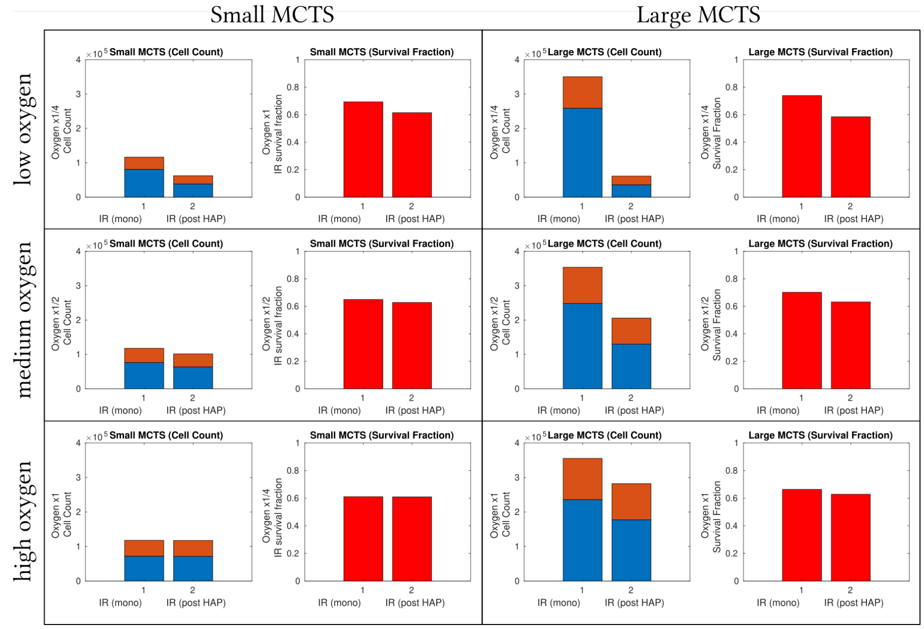

Fig D. Treatment responses of radiotherapy in various MCTSs when either (1) an IR monotherapy dose is administered at T_0+48 hours or (2) IR is given at T_0+48 hours following a prior HAP dose at time T_0. Complement to Fig 13 in the main manuscript. Cells are removed from the lattice instantaneously after the lethal event occurred.

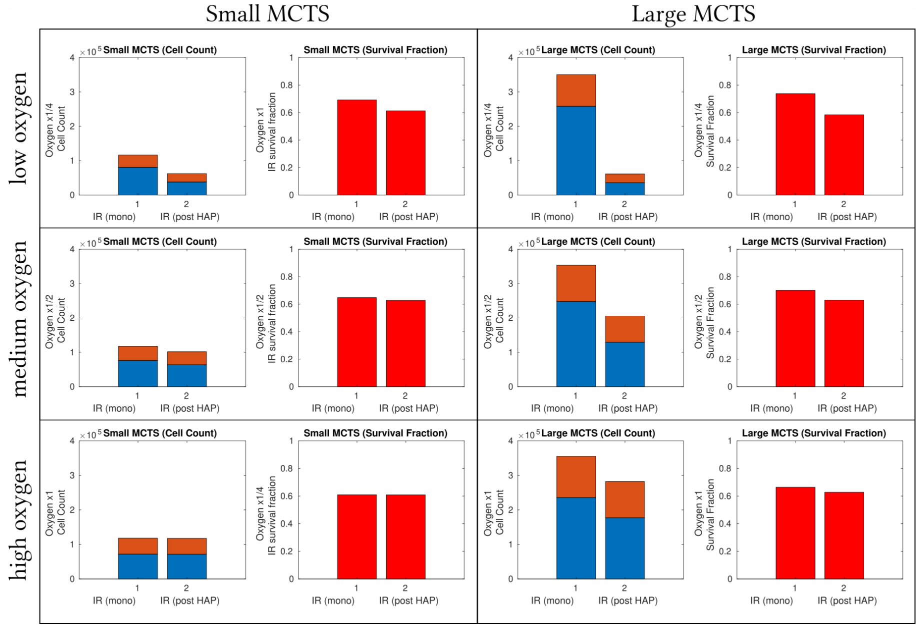

Fig E. Treatment responses of radiotherapy in various MCTSs when either (1) an IR monotherapy dose is administered at T_0+48 hours or (2) IR is given at T_0+48 hours following a prior HAP dose at time T_0. Complement to Fig 13 in the main manuscript. Cells are removed from the lattice after a time corresponding to their doubling time (τ_i) post the lethal event occurred.

S1 Text-3: Complement to Fig 15

As a complement to Fig 15, and the investigation concerning how the intra-tumoural oxygen landscape impacts HAP efficacy, we here introduce three more *in silico* multicellular tumour spheroids (MCTSs): MCTS C, D and E, in addition to MCTS A and B introduced in Fig 14 in the main manuscript. The MCTSs are visualised in Fig F, where all MCTSs contain the same number of hypoxic (pO_2 = 1 mmHg) and well-oxygenated (pO_2 = 100 mmHg) cells before treatment commences. The cell count over time when each of the MCTSs are subjected to a HAP dose at zero hours is available in Fig G, which illustrates that the oxygen landscape impacts how many cells survive the HAP treatment.

S1 Text-4: Pseudo-code flowchart

A diagrammatic representation of the code used in this study is provided in Fig H.

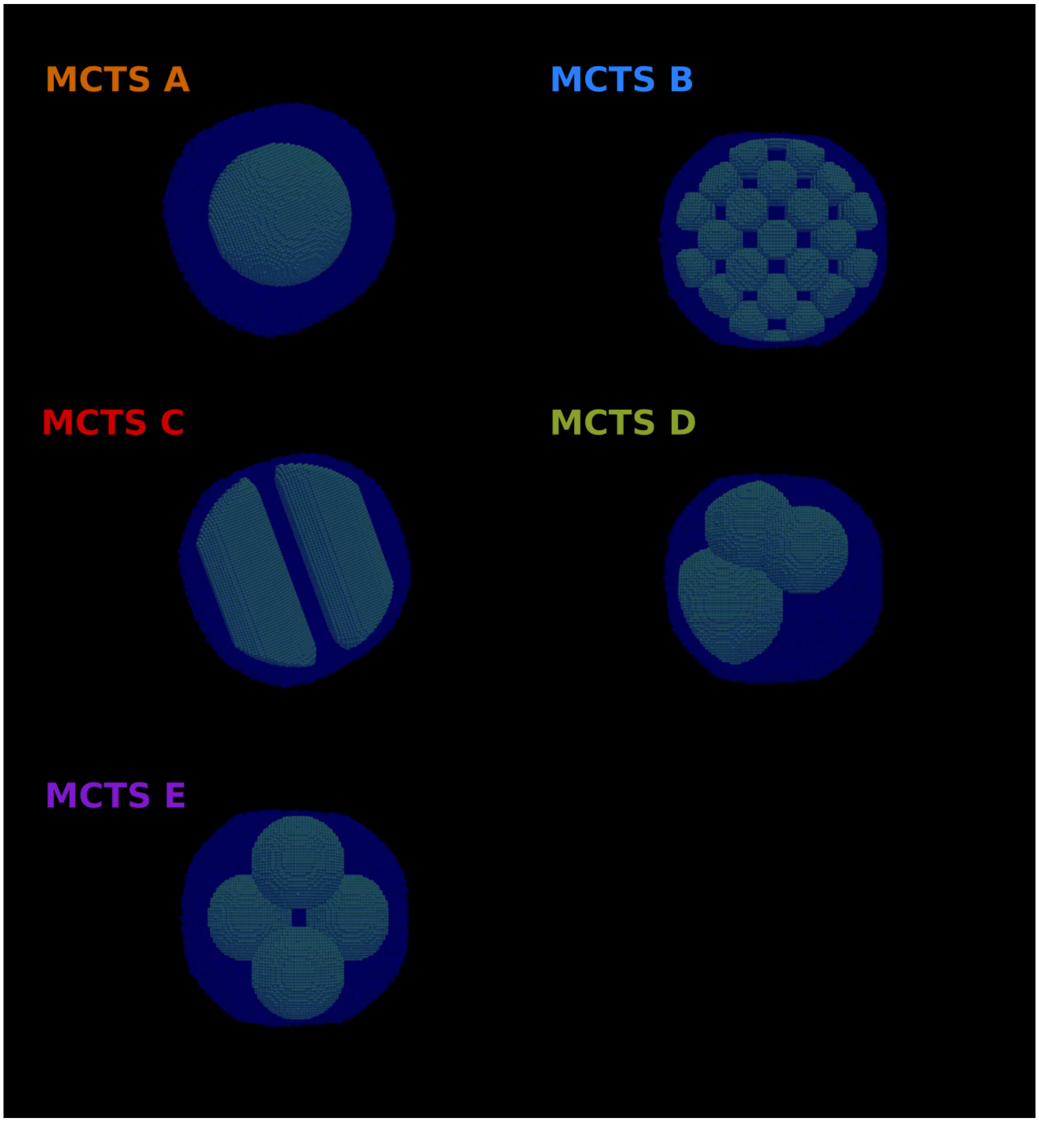

Fig F. MCTSs A-E all comprise the same number of hypoxic and well-oxygenated cells, but the hypoxic cells are clustered in different ways in the various MCTSs. Green cells are hypoxic (pO_2 = 1 mmHg) and blue cells are well-oxygenated (pO_2 = 100 mmHg).

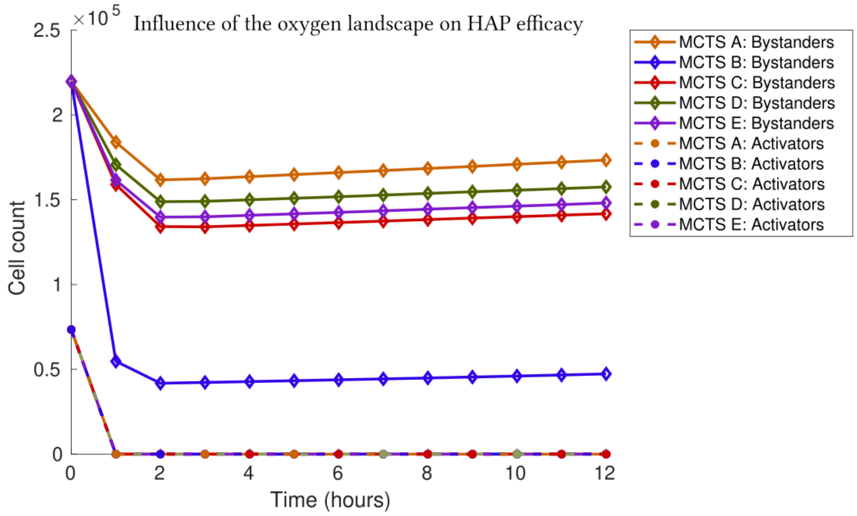

Fig G. Cell count over time when MCTSs A-E are subjected to a HAP dose at zero (0) hours. Mean values, based on 10 *in silico* runs, are shown. The resulting standard deviations are less than 0.5% of the means and hence not visible in the plot.

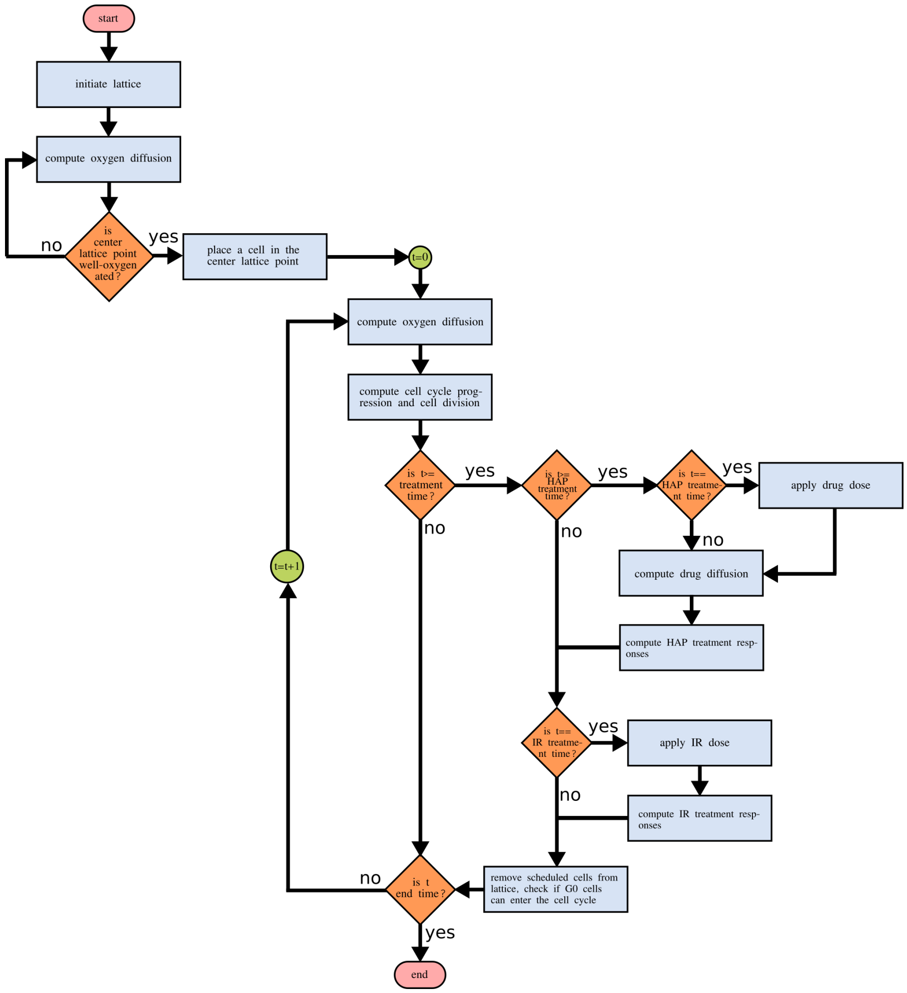

Fig H. A pseudo-code flowchart describing the basic structure of the *in silico* experiments. An in-house C++ code is used for model implementation.
